# Loss of chromosome 11p alleles in cultured cells derived from Wilms' tumours.

**DOI:** 10.1038/bjc.1989.213

**Published:** 1989-07

**Authors:** K. W. Brown, A. P. Shaw, V. Poirier, S. J. Tyler, P. J. Berry, M. G. Mott, N. J. Maitland

**Affiliations:** Department of Pathology, Medical School, University Walk, Bristol, UK.

## Abstract

**Images:**


Br. J. Cancer (1989), 60, 25–29 © The Macmillan Press Ltd., 1989

### Loss of chromosome 11p alleles in cultured cells derived from Wilms' tumours

K.W. Brown[1], A.P.W. Shaw[1], V. Poirier[1], S.J. Tyler[1], P.J. Berry[2], M.G. Mott[2] & N.J. Maitland[1]

[1]*CLIC Research Unit, Department of Pathology, The Medical School, University Walk, Bristol BS8 1TD, UK; and* [2]*Bristol Royal Hospital for Sick Children, St Michael's Hill, Bristol BS8 8BJ, UK.*

**Summary** Cell cultures have been produced from five Wilms' tumours. All cultures had a finite lifespan and a pattern of antigen expression which indicated that the cells were derived from the differentiated components of the tumours. No cells showed any of the expected characteristics of the putative Wilms' tumour stem cell. Nevertheless, in both cases where the original tumours showed a loss of heterozygosity at chromosome 11p alleles, the cultured cells also demonstrated a loss of heterozygosity. Thus these cell cultures definitely originated from Wilms' tumour tissue. The results demonstrate that cell cultures can be produced from the differentiated tissues present in Wilms' tumours and that these non-immortal cells show no 'transformed' phenotype, even though they possess the genetic changes present in the original tumour.

Wilms' tumour (nephroblastoma) is a malignant embryonal kidney tumour, and is one of the commonest solid tumours of childhood (Pochedly & Baum, 1984). There is now a great deal of evidence which suggests that the development of this tumour is associated with the loss of function of a recessive gene on the short arm of chromosome 11 (11p13) (Brodeur, 1984; Housman *et al.*, 1986; Porteous *et al.*, 1987; Shows *et al.*, 1986; Solomon, 1984; Weissman *et al.*, 1987). This often involves the loss or reduplication of part or all of the short arm of chromosome 11, which can be detected by examining the tumours for loss of heterozygosity at various polymorphic loci on chromosome 11 (Fearon *et al.*, 1984; Glaser *et al.*, 1986; Koufos *et al.*, 1984; Orkin *et al.*, 1984; Reeve *et al.*, 1984). We have used this methodology to examine cell lines which we have derived from several Wilms' tumours, in order to assess whether they were truly tumour-derived.

### Materials and methods

### *Cell culture*

Cultures were initiated from fresh samples of Wilms' tumours by mincing the tissue finely and then placing the fragments in plastic flasks, in Dulbecco's modified Eagle's medium, containing 15% fetal bovine serum, 10 ng ml$^{-1}$ epidermal growth factor, 1 $\mu$g ml$^{-1}$ hydrocortisone and 0.2 U m$^{-1}$ insulin. Mitomycin treated Swiss 3T3 cells (2–4 $\times 10^5$ cells per 25 cm$^2$ flask) were added as a feeder layer. When outgrowths had established, they were passaged routinely using trypsin/EDTA, at a 1:2 split ratio.

### *DNA analysis*

DNA was extractd from normal kidney tissue or lymphoblastoid cell lines (N), Wilms' tumour tissue (W) or cultured cells (C) using a guanidine isothiocyanate-density gradient centrifugation method, as described by Maitland *et al.* (1987). Ten microgram samples were digested with appropriate restriction endonucleases, electrophoresed on 1% agarose gels and blotted on to nylon membranes ('Hybond-N', Amersham International) using standard protocols (Maniatis *et al.*, 1982). Filters were prehybridised for 2 h at 45°C in 2 × SSC (0.3 M NaCl, 0.03 M sodium citrate, pH7), containing 33% formamide, 10 × Denhardt's solution (0.2% bovine serum albumin, 0.2% ficoll, 0.2% polyvinyl pyrollidone), 10% dextran sulphate, 0.5% sodium dodecyl sulphate (SDS), 2 mM EDTA and 100 $\mu$g ml$^{-1}$ denatured salmon sperm DNA (except when using minisatellite probes, where carrier DNA was omitted), and then hybridised overnight in the same solution containing a $^{32}$P-DNA probe labelled to high specific activity (about $10^9$ c.p.m. per $\mu$g) using the random-primed labelling method (Feinberg & Vogelstein, 1983; Amersham 'multiprime' kit). Filters were washed once in 2 × SSC at room temperature for 30 min, then in 2 × SSC, 0.5% SDS for 2 h at 65°C and finally in 0.1 × SSC for 30 min at room temperature, and then exposed to Hyperfilm-MP film (Amersham) at −70°C with intensifying screens.

The 11p probes used in this study were as follows: CAT:pSP65 (Boyd *et al.*, 1986), CALCA:phTB3 (Hoppener *et al.*, 1984), HBG:pHd3.2 (Old *et al.*, 1986), HBB:pPst$\beta$ (Old *et al.*, 1982), INS:pHINS310 (Bell *et al.*, 1981) and HRAS1:pEJ6.6 (Reeve *et al.*, 1984).

The multilocus minisatellite probe 6.3 was used for DNA fingerprinting (Jeffreys *et al.*, 1985).

### *Immunofluorescence*

Cultured cells were fixed in methanol/acetone (1:1, v/v) and then stained using indirect immunofluorescence (visualised by FITC-labelled anti-mouse immunoglobulins; Dako).

The monoclonal antibodies used were to: vimentin (Osborn *et al.*, 1984; Amersham International), keratin (a pan-epithelial anti-keratin monoclonal antibody; Leigh *et al.* (1985); Dako), desmin (Debus *et al.* (1983), Amersham), a neuroectodermal marker (UJ13A, Allan *et al.* (1983), kindly supplied by Mr S. Bourne, Frenchay Hospital, Bristol) and class 1 HLA (W632, Barnstable *et al.* (1978), Serotec).

### Results

Of a total of 14 Wilms' tumour samples, cultures have been derived from 11, and of these five have been studied in detail. The properties of these cells and the tumours from which they were derived are summarised in Tables I and II.

The morphology of the cells varied from fibroblastic (Figure 1a) to a more epithelial shape (Figure 1b). Two of the cell lines contained only vimentin intermediate filaments (Figure 1c and Table II), whereas two others expressed keratins as well as vimentin (Figure 1d and Table II) and one cell line expressed desmin in addition to vimentin (Figure 1e and Table II). All the cells tested expressed class 1 HLA, as shown by staining with the monoclonal antibody W632 (Figure 1f and Table II), whereas none of the cells tested stained positively for the 'neuroectodermal' marker

Correspondence: K.W. Brown.
Received 19 September 1988, and accepted in revised form, 21 February 1989.

**Table I** Wilms' tumours used to derive cell lines

| *Patient code* | *Tumour type* | *Tumour histology* | *Loss of 11p heterozygosity* |
|---|---|---|---|
| WT1 | Sporadic | Triphasic, muscle present | – |
| WT11 | Sporadic | Triphasic, prominent epithelial differentiation | + |
| WT4 | Sporadic | Blastema and stroma | – |
| WT5 | Sporadic | Prominent blastema with focal rosettes and epithelial differentiation | + |
| WT7 | Wilms'-aniridia | Triphasic with prominent stroma, including muscle | – |

**Table II** Properties of cell lines derived from Wilms' tumours

| *Patient code* | *Cell morphology* | *Maximum passage number* | *Immunofluorescence* Vimentin | Keratin | Desmin | UJ13A | Class 1 HLA |
|---|---|---|---|---|---|---|---|
| WT1 | Epithelial | 7 | + | + | – | ND | ND |
| WT11 | Epithelial | 8 | + | + | – | – | + |
| WT4 | Fibroblastic | 14 | + | – | – | ND | ND |
| WT5 | Fibroblastic | 6 | + | – | – | – | + |
| WT7 | Fibroblastic | 10 | + | – | + | – | + |

ND = not done.

**Table III** Chromosome 11p alleles in Wilms' tumours and cultured cells

| *Marker location enzyme* | | *CAT 11p13 SinI* | *CAT 11p13 KpnI* | *CAT 11p13 HaeIII* | *CAT 11p13 TaqI* | *CALCA 11p15.4 TaqI* | *HBG1 11p15.5 HindIII* | *HBG2 11p15.5 HindIII* | *HBB 11p15.5 SinI* | *HBB 11p15.5 BamHI* | *INS 11p.15.5 PvuII or HinfI* | *HRAS1 11p15.5 BamHI* | *HRAS1 11p15.5 MspI* | *HRAS1 11p15.5 TaqI* |
|---|---|---|---|---|---|---|---|---|---|---|---|---|---|---|
| WT1 | N | – | – | – | – | – | – | 1,2 | 1,2 | – | 1,2 | 1,2 | 1,2 | 1,2 |
| | T | – | – | – | – | – | – | 1,2 | 1,2 | – | 1,2 | 1,2 | 1,2 | 1,2 |
| WT11 | N | 1,2 | 1,2 | ND | 1,2 | – | – | – | – | ND | – | ND | 1,2 | 1,2 |
| | T | 1,2 | 1,2 | ND | 1,2 | – | – | – | – | ND | – | ND | 2 | 2 |
| | C | 1,2 | 1,2 | ND | 1,2 | – | – | – | – | ND | – | ND | 2 | 2 |
| WT4 | N | – | 1,2 | – | 1,2 | – | – | 1,2 | – | – | – | – | – | – |
| | T | – | 1,2 | – | 1,2 | – | – | 1,2 | – | – | – | – | – | – |
| WT5 | N | – | 1,2 | – | 1,2 | – | – | 1,2 | 1,2 | – | 1,2 | 1,2 | 1,2 | 1,2 |
| | T | – | 1,1 | – | 1,1 | – | – | 2,2 | 1,1 | – | 2,2 | 2,2 | 2,2 | 2,2 |
| | C | ND | ND | ND | 1,1 | ND | ND | ND | ND | – | 2,2 | 2,2 | ND | 2,2 |
| WT7 | N | 1,2 | – | 1,2 | – | – | – | – | – | 1,2 | – | 1,2 | 1,2 | 1,2 |
| | T | 1,2 | – | 1,2 | – | – | – | – | – | 1,2 | – | 1,2 | 1,2 | 1,2 |
| | C | ND | ND | ND | ND | ND | – | – | ND | 1,2 | ND | 1,2 | 1,2 | 1,2 |

1, larger allele; 2, smaller allele; –, non-informative, i.e. normal tissue was homozygous; ND, not done or unreadable (alleles too close together); N, normal tissue; T, Wilms' tumour tissue; C, Wilms' tumour cultures.

recognised by the monoclonal antibody UJ13A (data not shown; Table II). In all cases, the cells had a finite lifespan; surviving only six to 14 passages in culture (Table II). They all grew as monolayer cultures and showed no morphological evidence of a transformed phenotype.

All of the cell lines were derived from patients who were heterozygous for various polymorphic 11p loci, detected using probes to catalase (CAT), calcitonin (CALCA), beta (HBB) and gamma-globin (HBG1 and HBG2), insulin (INS) and c-Ha-*ras* 1 (HRAS1) and appropriate restriction endonuclease digestions (Table III). Three patients showed no loss of heterozygosity at any of these loci in their tumour DNA when compared to their normal DNA (WT1, 4 and 7, Table III). Since these tumours could not be distinguished from the normal tissue by allele analysis, the alleles present in the cultured cells were only investigated in one representative case (WT7) and found to be identical at all six enzyme/probe combinations examined (Table III).

Two tumours (WT5 and WT11) showed loss of 11p alleles (Figure 2a and Table III); in the case of WT5, the tumour showed a loss of heterozygosity at all informative 11p loci, whereas WT11 showed loss of 11p15 but not 11p13 allelles (Table III). Densitometric analysis of the autoradiographs demonstrated that WT5 became homozygous for the retained allelles, but that WT11 became hemizygous (data not shown).

At all loci examined in the cultured cells from WT5 and WT11, there was an identical pattern of loss of heterozygosity as that found in the original tumour tissue (Figure 2a and Table III). This was shown for four informative probe/enzyme combinations for WT5 and two combinations for WT11. In addition, WT11 tumour cells retained heterozygosity at 11p13, as found in the tumour tissue (Table III, three enzyme/probe combinations).

The patient origin of these cell lines was confirmed by 'DNA fingerprinting' (Figure 2b), using a multilocus probe to hypervariable minisatellite sequences (Jeffreys *et al.*, 1985). Thus these two cell lines (WT5 and 11) were genotypically identical, at 11p loci, to the tumours from which they originated, and were therefore definitely derived from Wilms' tumour tissue.

### Discussion

Wilms' tumour is thought to develop from the metanephric blastema, an embryonic cell type, which is induced by the developing ureteric bud to differentiate into both the epithelial

**Figure 1** Morphology and antigen expression of cells cultured from Wilms' tumours: (**a**) and (**b**) phase contrast micrographs of cells from WT7 and WT11 respectively. (**c**) to (**f**) immunofluorescence micrographs: (**c**) WT5 cells stained with anti-vimentin, (**d**) WT11 cells stained with anti-keratin, (**e**) WT7 cells stained with anti-desmin and (**f**) WT11 cells stained with anti-class 1 HLA (W632). Bar = 20 μm.

and stromal components of the mature kidney (Machin, 1984; Mierau & Beckwith, 1987; Ekblom, 1981). Wilms' tumours classically contain three components (normally referred to as a triphasic histology): (1) undifferentiated blastema, (2) epithelial elements and (3) stroma (of which striated muscle often forms a part); the latter two being derived from the former (by analogy with normal kidney development). Thus it is likely that the blastema represents the malignant stem cell compartment of the tumour, and the stromal and epithelial elements are differentiated derivatives of the blastema.

Immunohistochemical studies using antibodies to intermediate filament proteins have defined some of the *in vivo* characteristics of the three components of Wilms' tumour: (1) the blastema cells always contain vimentin and may express keratins weakly in some areas, (2) the epithelial cells contain only keratins and (3) the stromal cells contain vimentin, and also desmin where striated muscle is present (Altmannsberger *et al.*, 1984; Denk *et al.*, 1985; Kahn *et al.*, 1983; Yeger *et al.*, 1985; Berry *et al.*, unpublished results). Additionally, it has been shown that the blastema cells do not express class 1 HLA, whereas the differentiated cells do (Borthwick *et al.*, 1988; Shaw *et al.*, 1988). In contrast, the antibody UJ13A mainly stains the blastema in Wilms' tumours (Berry *et al.*, unpublished results).

Assuming that the various cell types maintain these characteristics in culture, it seems likely that the cell lines described in this paper are derived from the epithelial (in the case of WT1 and 11) and stromal (in the case of WT4, 5 and 7) elements of their respective tumours, and none show the expected characteristics of blastema cells. One of the strongest pieces of evidence in favour of this conclusion is the finding that none of the cell lines tested stained positively with the monoclonal antibody UJ13A (Table II). UJ13A detects a fetal antigen originally described as a neuro-ectodermal marker, but which is also expressed by blastema cells and a few epithelial tubules in both fetal kidney and Wilms' tumour (Allan *et al.*, 1983; Berry *et al.*, unpublished

**Figure 2** Loss of heterozygosity at chromosome 11p alleles in cells cultured from Wilms' tumours. (**a**) The polymorphic patterns shown were obtained using a HRAS1 probe (see Methods) on Bam-HI digests for patient WT5 and on MSP-I digests for patient WT11. Allelic bands are numbered as in Table III. Allele 1 was lost in the tumour and culture DNA in both cases. (**b**) DNA 'fingerprints' were produced by probing blots of Hinf-I digests with the multilocus minisatellite probe 6.3 (see Methods). The positions of molecular weight markers are shown on the right of each set of tracks. Only the regions of the blots containing the polymorphic bands are shown in (**a**). N, normal tissue; W, Wilms' tumour tissue; C, Wilms' tumour cultures.

results). Recent results have shown that UJ13A reacts with the neural cell adhesion molecule (N-CAM) (K. Patel & J. Kemshead, personal communication), and reports from other workers have confirmed that N-CAM is expressed in Wilms' tumours (Roth *et al.*, 1988) and in the undifferentiated nephrogenic mesenchyme (blastema) in mouse embryos (Klein *et al.*, 1988). In addition, some of our cell lines (WT4, 5 and 7) have previously been shown not to express detectable levels of N-*myc* RNA (Shaw *et al.*, 1988), although N-*myc* is expressed at high levels in the blastema cells in Wilms' tumours *in vivo* (Shaw *et al.*, 1988).

Our demonstration of the loss of 11p allelles in the cultured cells derived from two tumours (Figure 2a and Table III), provides unequivocal evidence that these cell cultures (WT5 and WT11) were truly tumour-derived. Although we have not been able to prove that the cell lines obtained from tumours which showed no loss of heterozygosity were tumour-derived, it is likely that these cells were also derived from the differentiated parts of the tumours, since all of them express class 1 HLA and none stain positively with UJ13A. Preliminary cytogenic studies on one of these latter cell lines (WT7) have shown the presence of a marker chromosome (data not shown) indicating that these cells were also tumour-derived.

Three of our cell lines were tested for their ability to produce tumours in athymic nude mice, with negative results (WT1, 7 and 11; approximately $10^7$ cell injected subcutaneously per mouse, tumour-free periods between 112 and 225 days). A lack of material (due to the finite lifespan of the cells) prevented a full study of the possible tumorigenic potential of all the cell lines. However, we are not aware of any cases in which human cells with a finite culture lifespan have formed malignant tumours in nude mice.

The failure to establish permanent cell lines from Wilms' tumours is consistent with many other earlier reports (reviewed by Hard, 1984), and to the recent results reported by Fraizer *et al.* (1987), who concluded that their non-immortal cell lines were tumour-derived on the basis of abnormal karyotypes. Our results and those of Fraizer *et al.* (1987) clearly show that the derivation of cells resembling normal kidney cells from Wilms' tumours does not necessarily represent a contamination with normal kidney, as proposed by others (Hard, 1984), but probably represents the growth of cells from the non-malignant, differentiated parts of the tumours.

Very few permanent cell lines have been established from Wilms' tumours (Hard, 1984), the most recent being a desmin-positive non-tumorigenic cell line, derived from a Wilms'-aniridia patient possessing a deletion on the short arm of chromosome 11 (Kumar *et al.*, 1987). Interestingly, one of our cell lines was derived from a Wilms'-aniridia patient with an 11p deletion (WT7), and this line was desmin-positive (Figure 1e and Table II), presumably originating from the differentiated striated muscle component often found in Wilms' tumours. However, our cell line did not establish in culture to form an immortal cell line.

The results described in this paper clearly demonstrated that the epithelial and stromal components of Wilms' tumour can be cultured to give non-immortal cell lines, which are nevertheless genotypically identical to their parental tumours. Paradoxically, the inability to culture the stem cell component of Wilms' tumours may indicate a strong potential for developing non-cytotoxic methods for treating these and similar embryonal tumours, since conventional culture conditions clearly select against the malignant cells in the tumours (even when using feeder layers and added growth factors, as in this paper).

Preliminary studies in our laboratory have shown that cells with some of the antigenic properties expected of the Wilms' tumour blastema (UJ13A-positive) are found in early primary cultures. However, these cells do not proliferate and can no longer be detected after 7 days under standard culture conditions. We suggest that there may be factors in serum which inhibit the proliferation and/or induce the differentiation of the blastema cells, since UJ13A-positive cells show a far longer survival in serum-free media (unpublished observations). Interestingly, Garvin *et al.* (1987) have recently described a serum-free culture system which allows the proliferation of putative blastema cells for six to 12 passages. Similarly, our preliminary attempts to culture Wilms' tumours (two samples) in serum-free medium have only given an extended survival of 'blastema' cells, without the formation of immortal cell lines. Clearly a full understanding of the biology of Wilms' tumour requires the development of suitable conditions for the routine culture of the blastema stem cell.

Some of the cell lines described in this paper are undoubtdly derived from Wilms' tumours, since they are genotypically identical to their parental tumours. These cells can be immortalised by SV40 and have selectable markers introduced into them (Poirier *et al.*, 1988). Such cell lines may provide an important long-term source of material for the study of the genetic alterations which lead to the development of Wilms' tumour, for example, by use in cell fusion experiments.

The authors thank Mrs J. Gilbert and Ms J. McRill for the typing, Mr C. Jeal and Mrs S. Hagin for the photography and Dr C. Paraskeva for his critical reading of the manuscript. The DNA probes used in this study were kindly supplied by Drs A. Jeffreys, N. Hastie and N. Teich and Professor R. Williamson. This work was supported by the Cancer and Leukaemia in Childhood Trust (CLIC). K.W.B. and V.P. are CLIC Research Fellows.

### References

ALLAN, P.M., GARSON, J.A., HARPER, E.I. and 4 others (1983). Biological characterization and clinical application of a monoclonal antibody recognizing an antigen restricted to neuroectodermal tissues. *Int. J. Cancer*, **31**, 591.

ALTMANNSBERGER, M., OSBORN, M., SCHAFER, H., SCHAUER, A. & WEBER, K. (1984). Distinction of nephroblastomas from other childhood tumours using antibodies to intermediate filaments. *Virchows Arch.* (*Cell Pathol.*), **45**, 113.

BARNSTABLE, C.J., BODMER, W.F., BROWN, G. and 4 others (1978). Production of monoclonal antibodies to group A erythrocytes, HLA and other human cell surface antigens – new tools for genetic analysis. *Cell*, **14**, 9.

BELL, G.I., KARAM, J.H. & RUTTER, W.J. (1981). Polymorphic DNA region adjacent to the 5′ end of the human insulin gene. *Proc. Natl Acad. Sci. USA*, **78**, 5759.

BORTHWICK, G.M., HUGHES, C., HOLMES, C.H., DAVIS, S.J. & STIRRAT, G.M. (1988). Expression of class I and II major histocompatibility complex antigens in Wilms' tumour and normal developing human kidney. *Br. J. Cancer*, **58**, 753.

BOYD, P., VAN HEYNINGEN, V., SEAWRIGHT, A., FEYKETE, G. & HASTIE, N. (1986). Use of catalase polymorphisms in the study of sporadic aniridia. *Hum. Genet.*, **73**, 171.

BRODEUR, G.M. (1984). Genetic and cytogenic aspects of Wilms' tumour. In *Wilms' Tumor. Clinical and Biological Manifestations*, Pochedly, C. & Baum, E.S. (eds) p. 125. Elsevier: New York.

DEBUS, E., WEBER, K. & OSBORN, M. (1983). Monoclonal antibody to desmin, the muscle-specific intermediate filament protein. *EMBO J.*, **2**, 2305.

DENK, H., WEYBORA, W., RATSCHER, M., SOHAR, R. & FRANKE, W.W. (1985). Distribution of vimentin, cytokeratins, and desmosomal-plaque proteins in human nephroblastoma as revealed by specific antibodies: co-existence of cell groups of different degrees of epithelial differentiation. *Differentiation*, **29**, 88.

EKBLOM, P. (1981). Determination and differentiation of the nephron. *Med. Biol.*, **59**, 139.

FEARON, E.R., VOGELSTEIN, B. & FEINBERG, A.P. (1984). Somatic deletion and duplication of genes of chromosome 11 in Wilms' tumours. *Nature*, **309**, 176.

FEINBERG, A.P. & VOGELSTEIN, B. (1983). A technique for radiolabelling DNA restriction endonuclease fragements to high specific activity. *Anal. Biochem.*, **132**, 6.

FRAIZER, G.E., BOWEN-POPE, D.F. & VOGEL, A.M. (1987). Production of platelet-derived growth factor by cultured Wilms' tumour cells and fetal kidney cells. *J. Cell. Physiol.*, **133**, 169.

GARVIN, A.J., SULLIVAN, J.L., BENNETT, D.D., STANLEY, W.S., INABNETT, T. & SENS, D.A. (1987). The *in vitro* growth, heterotransplantation and immunohistochemical characterization of the blastemal component of Wilms' tumour. *Am. J. Pathol.*, **129**, 353.

GLASER, T., LEWIS, W.H., BRUNS, G.A.P. and 8 others (1986). The Beta-subunit of follicle-stimulating hormone is deleted in patients with aniridia and Wilms' tumour, allowing a further definition of the WAGR locus. *Nature*, **321**, 881.

HARD, G.C. (1984). Tumor biology: in vitro culture and transplantation models of Wilms' tumor. In *Wilms' Tumor. Clinical and Biological Manifestations*, Pochedly, C. & Baum, E.S. (eds) p. 191. Elsevier: New York.

HOPPENER, J., STEENBERGH, P., ZANDBERG, J. and 5 others (1984). Localization of the polymorphic calcitonin gene on chromosome 11. *Hum. Genet.*, **66**, 309.

HOUSMAN, D.E., GLAZER, T., GERHARD, D.S., JONES, C., BRUNS, G.A.P. & LEWIS, W.H. (1986). Mapping of human chromosome 11: organisation of genes within the Wilms' tumor region of the chromosome. *Cold Spring Harbor Symp. Quant. Biol.*, **51**, 837.

JEFFREYS, A.J., WILSON, V. & THEIN, S.L. (1985). Individual-specific 'fingerprints' of human DNA. *Nature*, **316**, 76.

KAHN, H.J., YEGER, H., BAUMAL, R., THOM, H. & PHILLIPS, J.M. (1983). Categorization of pediatric neoplasms by immunostaining with antiprekeratin and antivimentin antisera. *Cancer*, **51**, 645.

KLEIN, G., LANGEGGER, M., GORIDIS, C. & EKBLOM, P. (1988). Neural cell adhesion molecules during embryonic induction and development of the kidney. *Development*, **102**, 749.

KOUFOS, A., HANSEN, M.F., LAMPKIN, B.C. and 4 others (1984). Loss of alleles at loci on chromosome 11 during the genesis of Wilms' tumour. *Nature*, **309**, 170.

KUMAR, S., HARRISON, C.J., HEIGHWAY, J., MARSDEN, H.B., WEST, D.C. & MORRIS-JONES, P. (1987). A cell line from Wilms' tumour with deletion in short arm of chromosome 11. *Int. J. Cancer*, **40**, 499.

LEIGH, I.M., PULFORD, K.A., RAMAEKERS, F.C.S. & LANE, E.B. (1985). Psoriasis: maintenance of an intact monolayer basal cell differentiation compartment in spite of hyperproliferation. *Br. J. Dermatol.*, **113**, 53.

MACHIN, G.A. (1984). Persistent renal blastema as a precursor of Wilms' tumor. In *Wilms' Tumor. Clinical and Biological Manifestations*, Pochedly, C. & Baum, E.S. (eds) p. 213. Elsevier: New York.

MAITLAND, N.J., COX, M.F., LYNAS, C., PRIME, S.S. & SCULLY, C. (1987). Detection of human papillomavirus DNA in biopsies of human oral tissue. *Br. J. Cancer*, **56**, 245.

MANIATIS, T., FRITSCH, E.F. & SAMBROOK, J. (1982). *Molecular Cloning. A Laboratory Manual.* Cold Spring Harbor Laboratory: New York.

MIERAU, G.W. & BECKWITH, J.B. (1987). Ultrastructure and histogenesis of the renal tumors of childhood: an overview. *Ultrastruct. Pathol.*, **11**, 313.

OLD, J.M., AYUB, H., WOOD, W.G., CLEGG, J.B. & WEATHERALL, D.J. (1982). Linkage analysis of nondeletion hereditary persistance of fetal hemoglobin. *Science*, **215**, 981.

OLD, J.M., HEATH, C., FITCHES, A. and 5 others (1986). Meiotic recombination between two polymorphic restriction sites within the $\beta$ globin gene cluster. *J. Med. Genet.*, **23**, 14.

ORKIN, S.H., GOLDMAN, D.S. & SALLAN, S.E. (1984). Development of homozygosity for chromosome 11p markers in Wilms' tumour. *Nature*, **309**, 172.

OSBORN, M., DEBUS, E. & WEBER, K. (1984). Monoclonal antibodies specific for vimentin. *Eur. J. Cell Biol.*, **34**, 137.

POCHEDLY, C. & BAUM, E.S. (1984). *Wilms' Tumor. Clinical and Biological Manifestations.* Elsevier: New York.

POIRIER, V., TYLER, S.J., BROWN, K.W., SHAW, A.P.W. & MAITLAND, N.J. (1988). SV40 transfection of human kidney epithelial cells and stability of chromosome 11. *Int. J. Cancer*, **42**, 887.

PORTEOUS, D.J., BICKMORE, W., CHRISTIE, S. and 9 others (1987). HRAS-1-selected chromosome transfer generates markers that colocalise aniridia- and genitourinary dysplasia-associated translocation breakpoints and the Wilms' tumour gene within band 11p13. *Proc. Natl Acad. Sci. USA*, **84**, 5355.

REEVE, A.E., HOUSIAUX, P.J., GARDNER, R.J.M., CHENINGS, W.E., GRINDLEY, R.M. & MILLOW, L.J. (1984). Loss of a Harvey ras allele in sporadic Wilms' tumour. *Nature*, **309**, 174.

ROTH, J., ZUBER, C., WAGNER, P. and 5 others (1988). Reexpression of poly(sialic acid) units of the neural cell adhesion molecule in Wilms' tumour. *Proc. Natl Acad. Sci. USA*, **85**, 2999.

SHAW, A.P.W., POIRIER, V., TYLER, S., MOTT, M., BERRY, P.J. & MAITLAND, N.J. (1988). Expression of the N-*myc* oncogene in Wilms' tumour and related tissues. *Oncogene*, **3**, 143.

SHOWS, T.B., DAVIS, L.M., QIN, S. & NOWAK, N.J. (1986). The chromosome 11 gene map: genes for growth and development, Wilms' tumor deletions and cancer chromosome breakpoints. *Cold Spring Harbor Symp. Quant. Biol.*, **51**, 867.

SOLOMON, E. (1984). Recessive mutation in aetiology of Wilms' tumour. *Nature*, **309**, 111.

WEISSMAN, B.E., SAXON, P.J., PASQUALE, S.R., JONES, G.R., GEISER, A.G. & STANBRIDGE, E.J. (1987). Introduction of a normal human chromosome 11 into a Wilms' tumour cell line controls its tumorigenic expression. *Science*, **236**, 175.

YEGER, H., BAUMAL, R., BAILEY, D., PAWLIN, G. & PHILLIPS, M.J. (1985). Histochemical and immunohistochemical characterization of surgically resected and heterotransplanted Wilms' tumor. *Cancer Res.*, **45**, 2350.


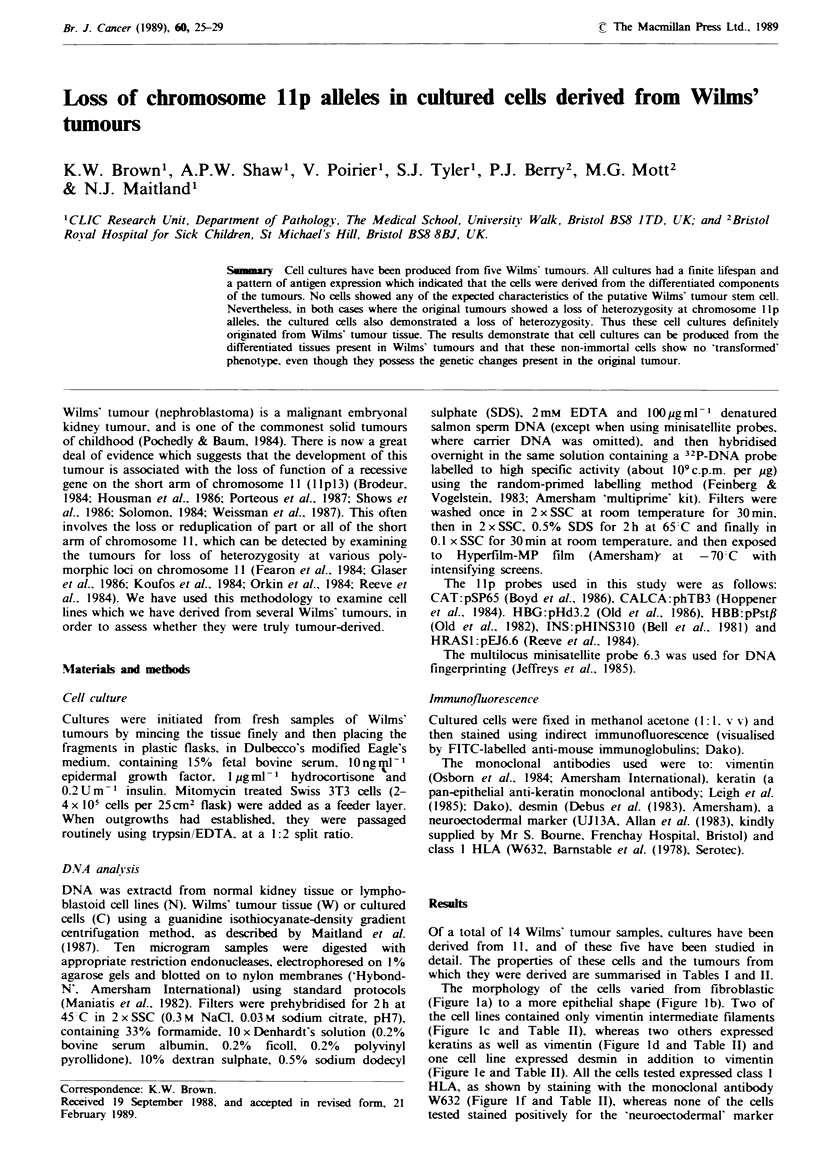

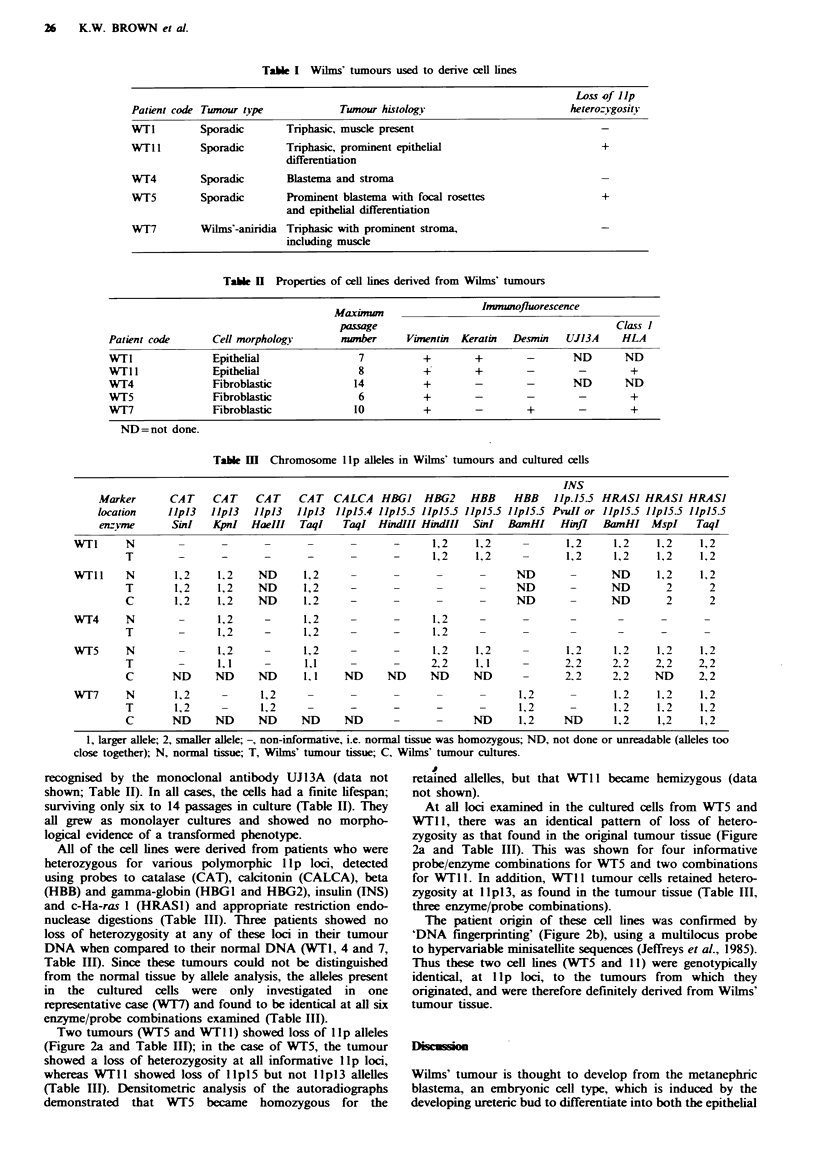

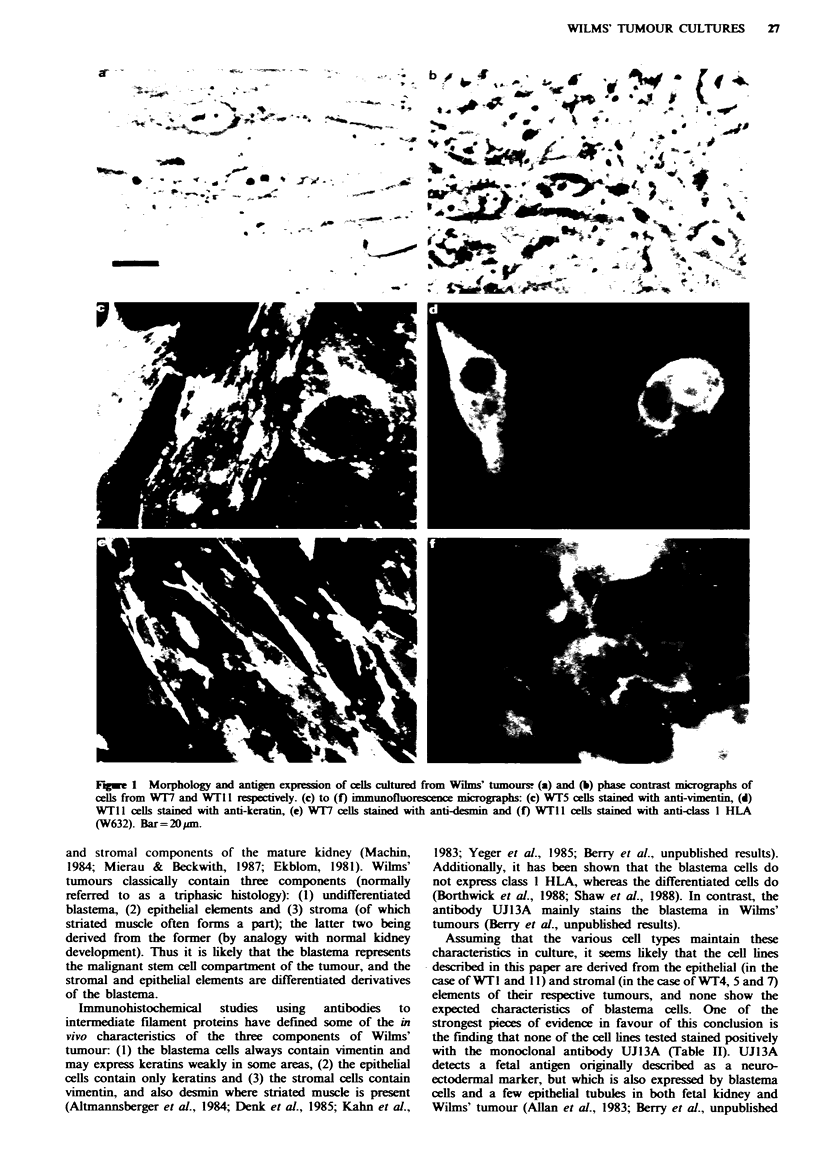

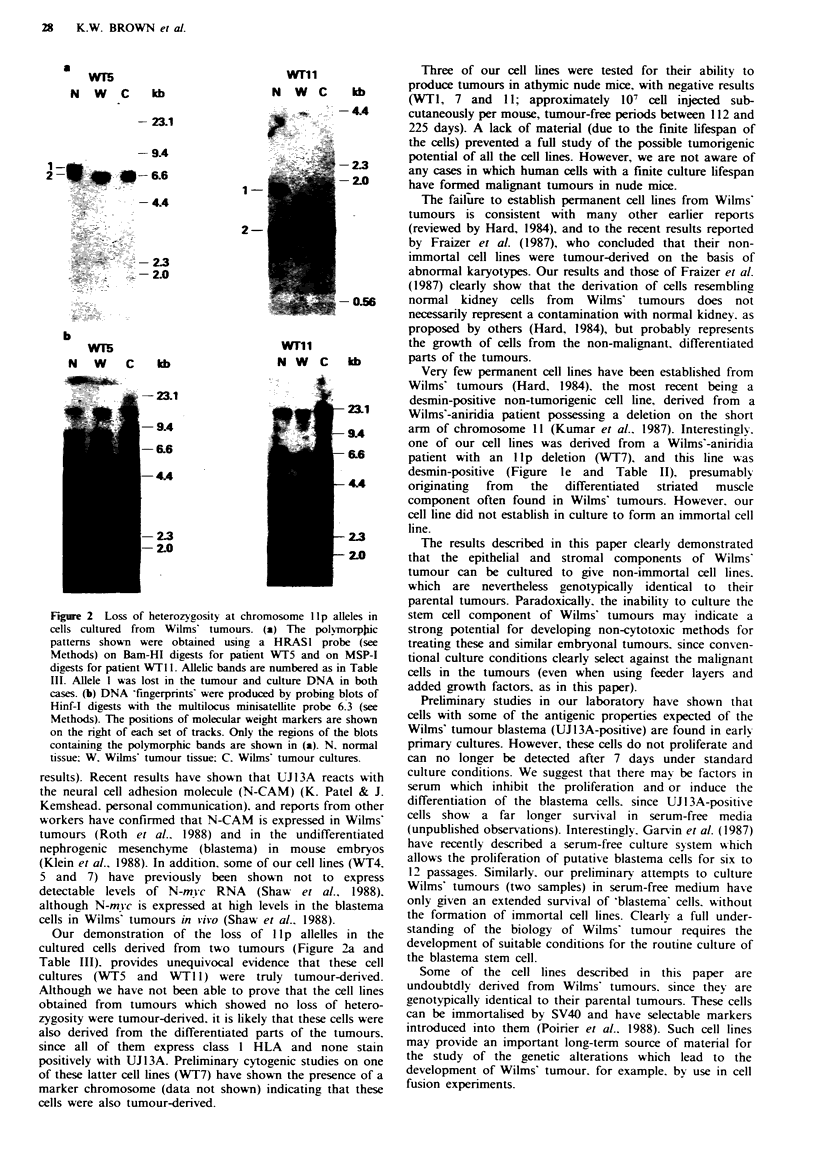

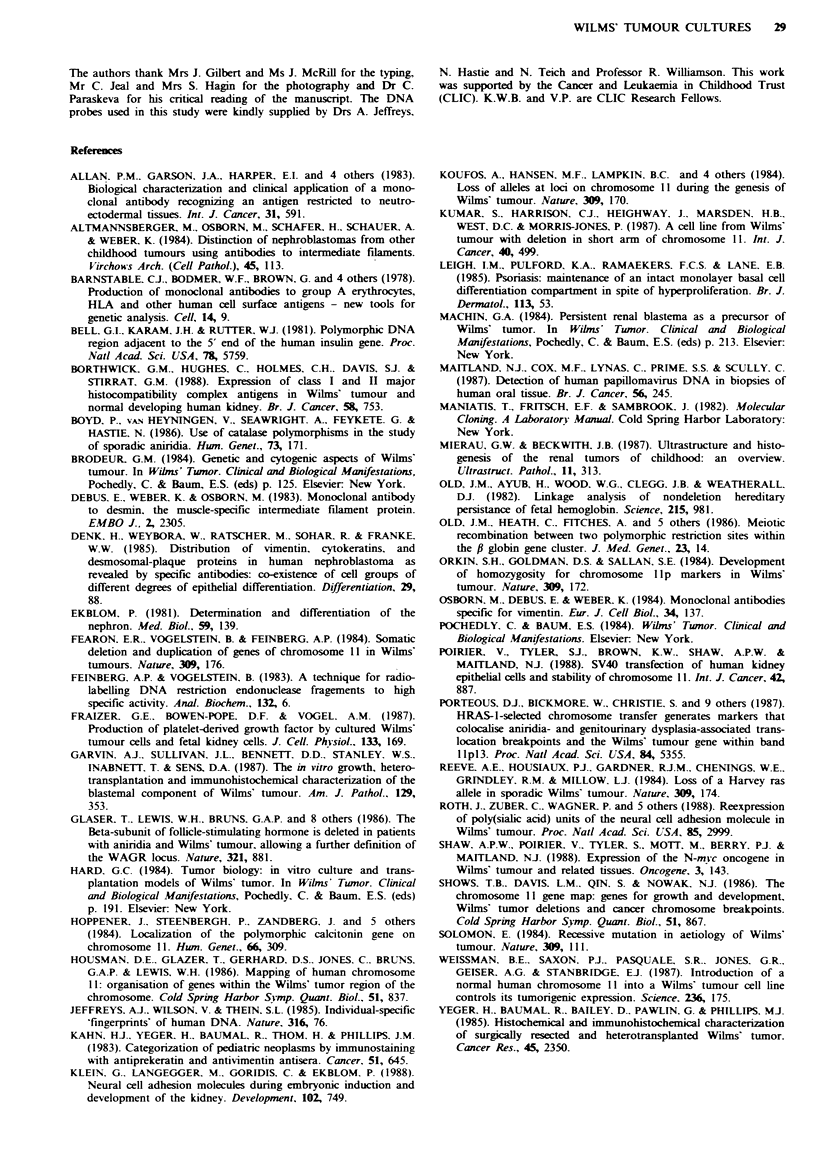


## References

[OCR_00633] Allan P. M., Garson J. A., Harper E. I., Asser U., Coakham H. B., Brownell B., Kemshead J. T. (1983). Biological characterization and clinical applications of a monoclonal antibody recognizing an antigen restricted to neuroectodermal tissues.. Int J Cancer.

[OCR_00639] Altmannsberger M., Osborn M., Schäfer H., Schauer A., Weber K. (1984). Distinction of nephroblastomas from other childhood tumors using antibodies to intermediate filaments.. Virchows Arch B Cell Pathol Incl Mol Pathol.

[OCR_00651] Bell G. I., Karam J. H., Rutter W. J. (1981). Polymorphic DNA region adjacent to the 5' end of the human insulin gene.. Proc Natl Acad Sci U S A.

[OCR_00656] Borthwick G. M., Hughes L., Holmes C. H., Davis S. J., Stirrat G. M. (1988). Expression of class I and II major histocompatibility complex antigens in Wilms' tumour and normal developing human kidney.. Br J Cancer.

[OCR_00662] Boyd P., van Heyningen V., Seawright A., Fekete G., Hastie N. (1986). Use of catalase polymorphisms in the study of sporadic aniridia.. Hum Genet.

[OCR_00672] Debus E., Weber K., Osborn M. (1983). Monoclonal antibodies to desmin, the muscle-specific intermediate filament protein.. EMBO J.

[OCR_00685] Ekblom P. (1981). Determination and differentiation of the nephron.. Med Biol.

[OCR_00689] Fearon E. R., Vogelstein B., Feinberg A. P. (1984). Somatic deletion and duplication of genes on chromosome 11 in Wilms' tumours.. Nature.

[OCR_00694] Feinberg A. P., Vogelstein B. (1983). A technique for radiolabeling DNA restriction endonuclease fragments to high specific activity.. Anal Biochem.

[OCR_00699] Fraizer G. E., Bowen-Pope D. F., Vogel A. M. (1987). Production of platelet-derived growth factor by cultured Wilms' tumor cells and fetal kidney cells.. J Cell Physiol.

[OCR_00704] Garvin A. J., Sullivan J. L., Bennett D. D., Stanley W. S., Inabnett T., Sens D. A. (1987). The in vitro growth, heterotransplantation, and immunohistochemical characterization of the blastemal component of Wilms' tumor.. Am J Pathol.

[OCR_00728] Housman D. E., Glaser T., Gerhard D. S., Jones C., Bruns G. A., Lewis W. H. (1986). Mapping of human chromosome 11: organization of genes within the Wilms' tumor region of the chromosome.. Cold Spring Harb Symp Quant Biol.

[OCR_00723] Höppener J. W., Steenbergh P. H., Zandberg J., Bakker E., Pearson P. L., Geurts van Kessel A. H., Jansz H. S., Lips C. J. (1984). Localization of the polymorphic human calcitonin gene on chromosome 11.. Hum Genet.

[OCR_00738] Kahn H. J., Yeger H., Baumal R., Thom H., Phillips J. M. (1983). Categorization of pediatric neoplasms by immunostaining with antiprekeratin and antivimentin antisera.. Cancer.

[OCR_00743] Klein G., Langegger M., Goridis C., Ekblom P. (1988). Neural cell adhesion molecules during embryonic induction and development of the kidney.. Development.

[OCR_00748] Koufos A., Hansen M. F., Lampkin B. C., Workman M. L., Copeland N. G., Jenkins N. A., Cavenee W. K. (1984). Loss of alleles at loci on human chromosome 11 during genesis of Wilms' tumour.. Nature.

[OCR_00755] Kumar S., Harrison C. J., Heighway J., Marsden H. B., West D. C., Jones P. M. (1987). A cell line from Wilms' tumour with deletion in short arm of chromosome II.. Int J Cancer.

[OCR_00759] Leigh I. M., Pulford K. A., Ramaekers F. C., Lane E. B. (1985). Psoriasis: maintenance of an intact monolayer basal cell differentiation compartment in spite of hyperproliferation.. Br J Dermatol.

[OCR_00771] Maitland N. J., Cox M. F., Lynas C., Prime S. S., Meanwell C. A., Scully C. (1987). Detection of human papillomavirus DNA in biopsies of human oral tissue.. Br J Cancer.

[OCR_00781] Mierau G. W., Beckwith J. B., Weeks D. A. (1987). Ultrastructure and histogenesis of the renal tumors of childhood: an overview.. Ultrastruct Pathol.

[OCR_00786] Old J. M., Ayyub H., Wood W. G., Clegg J. B., Weatherall D. J. (1982). Linkage analysis of nondeletion hereditary persistence of fetal hemoglobin.. Science.

[OCR_00796] Orkin S. H., Goldman D. S., Sallan S. E. (1984). Development of homozygosity for chromosome 11p markers in Wilms' tumour.. Nature.

[OCR_00801] Osborn M., Debus E., Weber K. (1984). Monoclonal antibodies specific for vimentin.. Eur J Cell Biol.

[OCR_00809] Poirier V., Tyler S. J., Brown K. W., Shaw A. P., Maitland N. J. (1988). SV40 transfection of human kidney epithelial cells and stability of chromosome 11.. Int J Cancer.

[OCR_00815] Porteous D. J., Bickmore W., Christie S., Boyd P. A., Cranston G., Fletcher J. M., Gosden J. R., Rout D., Seawright A., Simola K. O. (1987). HRAS1-selected chromosome transfer generates markers that colocalize aniridia- and genitourinary dysplasia-associated translocation breakpoints and the Wilms tumor gene within band 11p13.. Proc Natl Acad Sci U S A.

[OCR_00822] Reeve A. E., Housiaux P. J., Gardner R. J., Chewings W. E., Grindley R. M., Millow L. J. (1984). Loss of a Harvey ras allele in sporadic Wilms' tumour.. Nature.

[OCR_00827] Roth J., Zuber C., Wagner P., Taatjes D. J., Weisgerber C., Heitz P. U., Goridis C., Bitter-Suermann D. (1988). Reexpression of poly(sialic acid) units of the neural cell adhesion molecule in Wilms tumor.. Proc Natl Acad Sci U S A.

[OCR_00832] Shaw A. P., Poirier V., Tyler S., Mott M., Berry J., Maitland N. J. (1988). Expression of the N-myc oncogene in Wilms' tumour and related tissues.. Oncogene.

[OCR_00837] Shows T. B., Davis L. M., Qin S., Nowak N. J. (1986). The chromosome 11 gene map: genes for growth and development, Wilms' tumor deletions, and cancer chromosome breakpoints.. Cold Spring Harb Symp Quant Biol.

[OCR_00843] Solomon E. (1984). Recessive mutation in aetiology of Wilms' tumour.. Nature.

[OCR_00847] Weissman B. E., Saxon P. J., Pasquale S. R., Jones G. R., Geiser A. G., Stanbridge E. J. (1987). Introduction of a normal human chromosome 11 into a Wilms' tumor cell line controls its tumorigenic expression.. Science.

[OCR_00853] Yeger H., Baumal R., Bailey D., Pawlin G., Phillips M. J. (1985). Histochemical and immunohistochemical characterization of surgically resected and heterotransplanted Wilms' tumor.. Cancer Res.

